# A phosphorylation switch controls androgen biosynthesis in prostate cancer

**DOI:** 10.1172/JCI166499

**Published:** 2023-01-17

**Authors:** Yun Qiu

**Affiliations:** 1 Departments of Pharmacology, University of Maryland School of Medicine, Baltimore, Maryland, USA.; 2 Veterans Affairs Maryland Health Care System (VAMHCS), Baltimore, Maryland, USA.

## Abstract

Androgen biosynthesis enzyme 3β-hydroxysteroid dehydrogenase type 1 (3βHSD1) encoded by *HSD3B1* has emerged as a potential driver for therapeutic resistance in prostate cancer. Patients with homozygous *HSD3B1*(1245C) inheritance are intrinsically more resistant to currently available androgen/androgen receptor–targeting (AR-targeting) drugs. In this issue of the *JCI*, Li et al. present data on the regulation of 3βHSD1 phosphorylation and activity by tyrosine kinase BMX. Inhibition of BMX activity by genetic or pharmacologic approaches blocked androgen biosynthesis in prostate cancer cells and inhibited tumor growth in preclinical xenograft models. The findings provide insights into mechanisms underlying castration resistance in prostate cancer and reveal a potential strategy to circumvent therapeutic resistance in patients with homozygous *HSD3B1*(1245C) inheritance.

## Androgen biosynthesis and therapeutic resistance in prostate cancer

**Figure 1:**
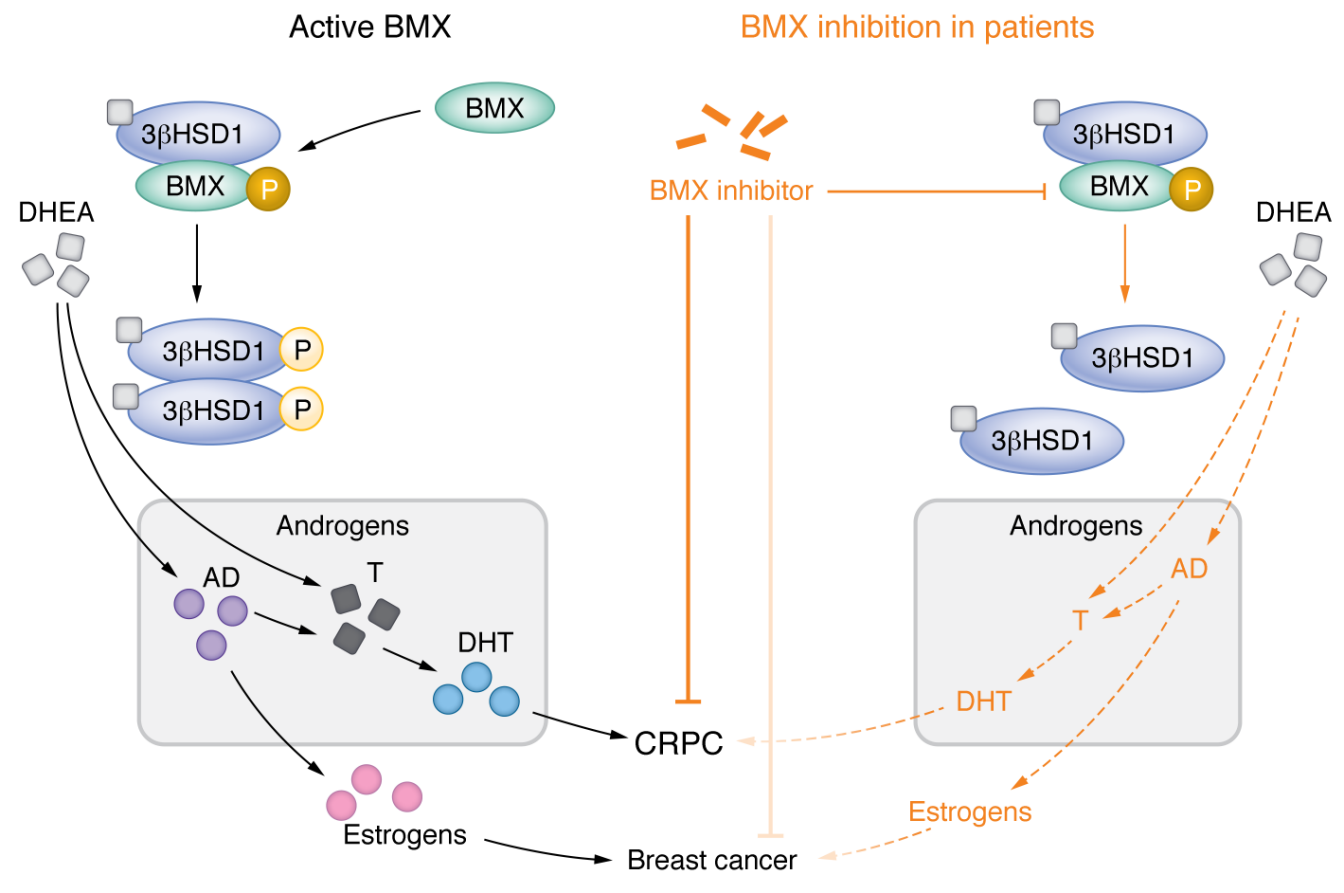
Targeting BMX regulation of androgen biosynthesis may circumvent therapeutic resistance in patients with prostate cancer. Patients with prostate cancer who are homozygous for *HSD3B1*(1245C) rapidly develop resistance to ADT. 3βHSD1 promotes the conversion of DHEA to DHT, which interacts with ARs to promote tumor growth and enable prostate cancer progression to CRPC. Li et al. (4) revealed a step in the androgen biosynthesis pathway involving 3βHSD1 and the protein tyrosine kinase BMX. In the presence of DHEA, BMX phosphorylated 3βHSD1 at Y344. Phosphorylated 3βHSD1 formed an active dimer to facilitate the conversion of DHEA to AD, which is a weak androgen hormone and intermediate in the biosynthesis of T and estrogens. T is subsequently converted to more active DHT in prostate cancer cells. Notably, inhibition of BMX interfered with androgen conversion in prostate cancer cells and tumor tissue derived from patients. Patients with homozygous *HSD3B1*(1245C) inheritance may benefit from treatments that target the BMX/3βHSD1 axis. This axis may also have relevancy in other sex-hormone-related cancers, such as breast cancer.

Androgens are driving forces for prostate cancer development and progression. Testosterone (T), primarily synthesized in testis, is one of the major circulating androgens. Upon androgen deprivation therapy (ADT), the level of circulating T substantially reduces, leading to regression of androgen-sensitive prostate cancer. However, the majority of patients will eventually develop castration resistant prostate cancer (CRPC) via multiple mechanisms. One of the principle mechanisms involves aberrant intracrine signaling due to increased activity of androgen-biosynthesis enzymes, such as the steroidogenic enzyme cytochrome P450 17A1 (CYP17A1), allowing prostate cancer cells to utilize dehydroepiandrosterone (DHEA), the adrenal androgen precursor for the synthesis of bioactive dihydrotestosterone (DHT). Abiraterone, used to treat metastatic prostate cancer, inhibits CYP17A1 activity and effectively blocks DHEA production. In addition to CYP17A1, another key rate-limiting androgen biosynthesis enzyme, 3β-hydroxysteroid dehydrogenase type 1 (3βHSD1) encoded by *HSD3B1*, has emerged as a potential driver for therapeutic resistance in prostate cancer (1). 3βHSD1 catalyzes the transition of DHEA to DHT or de novo synthesis from cholesterol. Previous studies revealed there are two common germline missense-encoding alleles *HSD3B1*(1245A) and *HSD3B1*(1245C) in prostate cancer patients. The *HSD3B1*(1245C) allele encodes for a more stable enzyme, driving more rapid DHT generation from extragonadal-precursor steroids. Multiple clinical studies have shown that patients with homozygous *HSD3B1*(1245C) inheritance — occurring in about 10% of the population — are intrinsically more resistant to ADT and first-line treatment with abiraterone or enzalutamide (1–3). Therefore, these patients may benefit from alternative therapeutics targeting androgen signaling. In this issue of the *JCI*, Li and colleagues identified tyrosine kinase BMX as a regulator of androgen biosynthesis through directly binding with and phosphorylating 3βHSD1 (4). They provided biochemical evidence that BMX formed a complex with 3βHSD1. In the presence of DHEA, BMX kinase activity was activated and 3βHSD1 was phosphorylated at tyrosine 344 (Y344). Phosphorylated 3βHSD1 formed an active dimer to promote the conversion of DHEA to DHT. Most importantly, the authors demonstrated that BMX blockage, either by siRNA or a pharmacologic inhibitor, blocked androgen biosynthesis in prostate cancer cells and cultured patient tissues. These findings provide insights into mechanisms underlying castration resistance in prostate cancer and reveal a potential strategy to circumvent therapeutic resistance in patients with homozygous *HSD3B1*(1245C) inheritance (Figure 1).

## Developing therapeutics that target the 3βHSD1/BMX axis

Previous phosphoproteomic analyses revealed the elevated activity of multiple tyrosine kinases in metastatic CRPC patient samples, suggesting that inhibition of these kinases benefit patients with CRPC (5). However, so far the efficacy of the targeted therapy for CRPC using kinase inhibitors has yet to be established due to the lack of reliable biomarkers for patient selection (6). BMX, also known as epithelial and endothelial tyrosine kinase (ETK), is a TEC family tyrosine kinase that is upregulated in response to castration and contributes to castration resistance in preclinical models (7, 8). The TEC family kinases are activated by various growth factors, cytokines, and matrix proteins (9). Li et al. provide evidence that extragonadal precursor steroids and 3βHSD1 together could also activate BMX in prostate cancer cells. In the presence of DHEA, an FDA approved Bruton’s tyrosine kinase (BTK) and BMX inhibitor, zanubrutinib, effectively blocked androgen receptor–positive (AR-positive) xenograft tumor growth in castrated mice (4). Given that patients with homozygous *HSD3B1*(1245C) inheritance have elevated 3βHSD1 activity, they may potentially benefit from BMX/BTK inhibitors. Investigators are conducting a multi-institutional phase 2 clinical trial to test the efficacy of combining the BMX/BTK inhibitor abivertinib with abiraterone in CRPC patients with *HSD3B1*(1245C) inheritance. If successful, the study may revitalize using other kinase inhibitors in combination therapy for CRPC. Meanwhile, it should be noted that, so far, BTK inhibitors are only successful in the treatment of lymphomas; their efficacy in solid tumors remains to be tested. Currently, all FDA-approved BTK inhibitors target the highly conserved kinase domain; therefore, they may display a broad-spectrum inhibition on all TEC family kinases. Given that TEC family kinases are important for normal B or T cell development and activation, adverse effects of these inhibitors on the normal immune system could be inevitable.

## Future opportunities

The current study has demonstrated a functional interaction between tyrosine kinase BMX and a key androgen synthesis enzyme, 3βHSD1. It is still intriguing how the 3βHSD1-BMX feed-forward loop is regulated in prostate cancer cells; the precise role of DHEA in activation of BMX remains unclear. Could direct binding of DHEA or 3βHSD1 to BMX via the PH domain induce a conformation change to mediate the feed-forward loop? Further understanding of the precise mechanisms underlying therapeutic resistance associated with *HSD3B1*(1245C) inheritance will allow us to identify more druggable targets. In addition to currently available FDA-approved small molecule inhibitors, BTK proteolysis-targeting chimeras (BTK-PROTACs) have emerged as a promising approach to improve the specificity and efficacy of BTK inhibitors (10). Similarly, more selective BMX-PROTACs could avoid some of the potential risks associated with small molecule inhibitors that target kinase domains. Furthermore, given that 3βHSD1 is also critical for conversion of extragonadal precursor steroids to estrogen and progesterone, the 3βHSD1/BMX axis may also play a role in other types of cancers, such as breast cancer. It is conceivable that these studies will lead to more effective treatments for steroid hormone–responsive cancers.

## Version 1 01/17/2023

Electronic publication
